# Antitumor Effect of a Polypeptide Fraction from *Arca subcrenata in Vitro* and *in Vivo*

**DOI:** 10.3390/md10122782

**Published:** 2012-12-11

**Authors:** Xianjing Hu, Liyan Song, Lijiao Huang, Qin Zheng, Rongmin Yu

**Affiliations:** 1 Biotechnological Institute of Chinese Materia Medica, Jinan University, Guangzhou 510632, China; E-Mails: huxj2003@163.com (X.H.); excellent19881007@163.com (L.H.); zhengqin027@163.com (Q.Z.); 2 College of Pharmacy, Jinan University, Guangzhou 510632, China

**Keywords:** *Arca subcrenata*, polypeptide, antitumor, S-180, H-22

## Abstract

*Arca subcrenata *Lischke is a marine traditional Chinese medicine. The study investigated the antitumor effects of P2, a polypeptide fraction from *A. subcrenata, *and its toxicity *in vitro* and *in vivo*. The results showed that P2 could inhibit the proliferation of seven tumor cell lines, especially in HeLa and HT-29 cell lines. The IC_50_ values were 11.43 μg/mL for HeLa and 13.00 μg/mL for HT-29 treated by P2 for 48 h. P2 had little cytotoxicity on normal liver cells (L-02). The maximum tolerated dose (MTD) of P2 on KM mice was 1000 mg/kg by i.p. or i.v. The tumor growth inhibitory ratios of P2 were 26.4%, 41.4% and 46.4% for H-22, and 34.0%, 45.8% and 60.1% for S-180 tumor-bearing mice. The results demonstrated that P2 might be a potential antitumor agent with high efficiency in dose-dependent and time-dependent manners and low toxicity.

## 1. Introduction

The ocean produces huge amounts of substance with various activities due to the environment with high salinity, high pressure, low temperature, low light intensity and an oligotrophic condition. In recent years, more and more scientists are diverting to exploit marine drugs for various kinds of bioactivities [1], including antioxidant [2,3], anti-influenza viral [4], anti-parasite [5], anti-hyperlipidemic activities [6], *etc.* Scientists discovered a number of compounds from marine environments, which exhibited good antitumor activity, such as Jaspine B [7] and fucoidan [8,9,10]. Recently, many polypeptides with antitumor activity were discovered from marine organisms, such as the officinal marine fish *Syngnathus acus *[11], the marine sponge *Discodermia calyx *[12] and the Indonesian marine sponge *Callyspongia aerizusa *[13].

*Arca subcrenata *Lischke is a marine invertebrate, which belongs to the Arcidae family under Phylum Mollusca, Class Lamellibranchiata. It is widely distributed in the neritic regions around China. A popular Traditional Chinese Medicine (TCM) called wa leng zi (Concha Arcae) is made of the shell of *A. subcrenata*. Furthermore, it is documented that the body of *A. subcrenata *could be useful in treatments of tumor, anemia, inflammation and dyspepsia [14]. Modern pharmacological studies revealed that *A. subcrenata *possessed various biological functions. Our previous report testified that the hydrolysates of *A. subcrenata* exhibited good antioxidant effects, including DPPH radical scavenging and hydrogen peroxide scavenging activities [15]. It was also demonstrated that *A. subcrenata *could evidently improve immunological function by promoting the thymus index and spleen index of normal mice and immunosuppression mice induced by cyclophosphamide *in vivo *[16], as well as stimulating mouse spleen lymphocyte proliferation *in vitro *[17,18]. 

*A. subcrenata *had been confirmed to inhibit the proliferation of several tumor cell lines [19,20]. However, no data demonstrated the *in vivo* antitumor activity and toxicity of *A. subcrenata *until now.

In the present study, the antitumor activity of P2, a polypeptide fraction from *A. subcrenata*, was investigated *in vitro* and *in vivo. *In order to evaluate the toxicity, acute toxicity testing was performed. This is the first report describing the *in vivo* antitumor activity and toxicity of *A. subcrenata*. 

## 2. Results

### 2.1. *In Vitro* Cytotoxicity Evaluation

#### 2.1.1. Effects of P2 on Human Tumor Cell Lines

**Figure 1:**
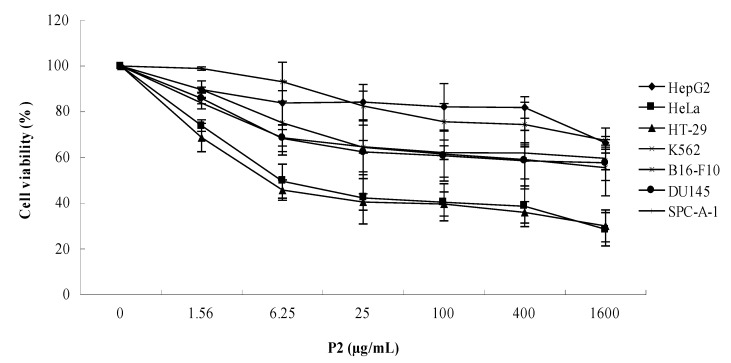
Effects of P2 on cell viability of human tumor cell lines. Seven tumor cell lines were treated by P2 at different concentrations for 48 h. Cell viability was determined by MTT assay. Data are presented as mean ± SD of three independent experiments.

To understand the effect of P2 on the proliferation of tumor cells, seven human tumor cell lines were chosen to evaluate the *in vitro* antitumor effects of P2. The sample was administered at different concentrations (0–1600 μg/mL) for 48 h, and cell viability was determined by MTT assay. The results (Figure 1) showed that P2 had the capability of anti-proliferation against seven human tumor cell lines, especially for HeLa and HT-29. The tumor cell lines of HeLa and HT-29 were much more sensitive to P2 than other tumor cell lines. 

#### 2.1.2. Cytotoxicity on Tumor Cells and Normal Cells

**Figure 2:**
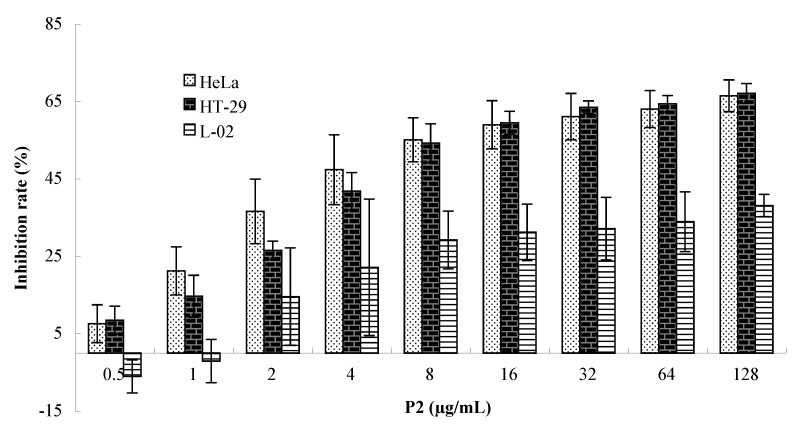
Selective cytotoxicity effects of P2 on HeLa and HT-29 cells compared with normal liver L-02 cells. Data are presented as mean ± SD of three independent experiments.

**Figure 3:**
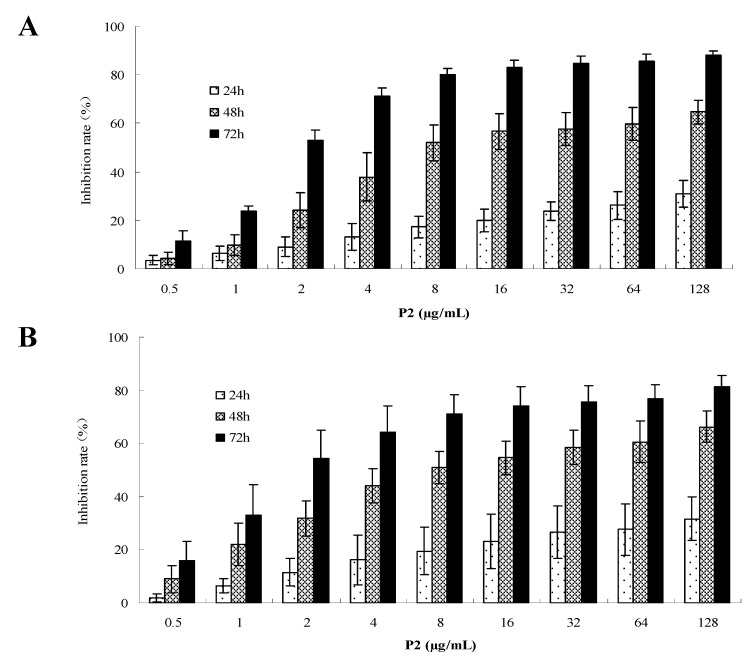
Cytotoxicity of P2 on HeLa (A) and HT-29 (B) cells in a dose- and time-dependent manner. The IC_50_ was calculated according to the inhibition rate by Graph Pad software. Data are presented as mean ± SD of three independent experiments.

**Figure 4:**
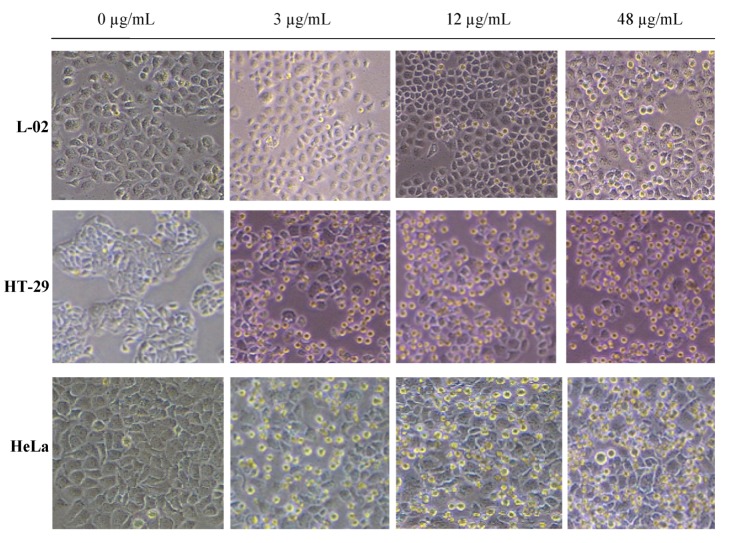
Cytotoxicity of P2 on cancer cells of HeLa and HT-29 and on normal liver L-02 cells.

In order to compare the cytotoxicity of P2 on tumor cells and normal cells, the human cervical cancer HeLa cells, colon cancer HT-29 cells and normal liver L-02 cells were exposed to a range of concentrations (0–128 μg/mL) of P2 for 48 h, and the cell growth inhibition was measured by MTT assay. The results showed that P2 had a high efficiency on HeLa and HT-29 cells, but low cytotoxicity on L-02 cells. The IC_50_ were 11.43 μg/mL for HeLa and 13.00 μg/mL for HT-29, and the highest inhibitory rate on L-02 cells was 38.10% among the concentrations (Figure 2). P2 exhibited good anti-proliferation activity on HeLa and HT-29 cells in dose-dependent and time-dependent manners (Figure 3). Bright field images (200×) of HeLa, HT-29 and L-02 cells treated with RPMI-1640 culture medium, and P2 is shown (Figure 4).

Bright field images are showing effects of P2 on HeLa, HT-29 and L-02 compared to RPMI-1640 culture medium control for each; magniﬁcation (200×, Nikon, Tokyo, Japan).

### 2.2. Acute Toxicity Testing

**Figure 5:**
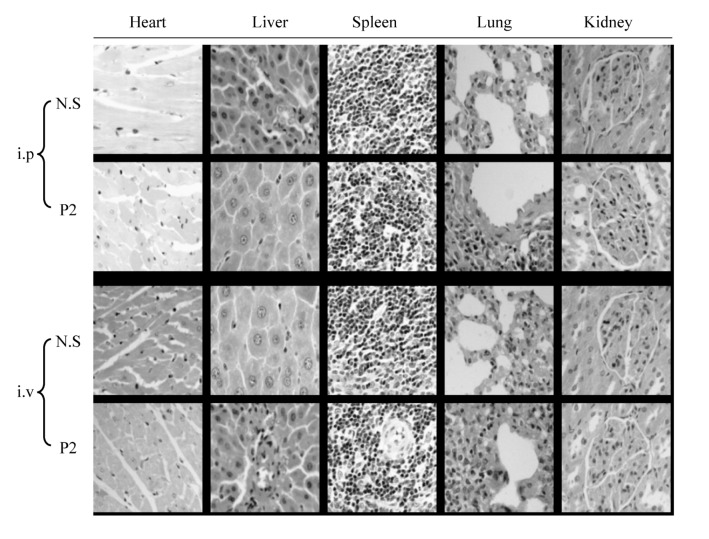
Histopathologic analysis by H & E staining of five kinds of organs of P2-treated mice comparing with control mice (normal saline).

For the acute toxicity testing, a total dosage of 1000 mg/kg of P2 was administered to the mice by intraperitoneal injection (i.p.) or intravenous injection (i.v.). The general behavior, body weight and fatality of treated mice were monitored daily for two weeks. As a result, the general behavior of P2 treated mice did not exhibit much variation for 14 days, except for lethargy on the first day after administration compared with the control mice. No fatality was observed during the experiment and no macroscopic lesions were seen on the organs of treated mice when being dissected after 14 days’ observation. The body weights of the treated mice had a bit of an alteration, but recovered five days after administration in both the i.p. group and i.v. group (Table 1). The results of histopathologic analysis revealed no gross morphologic difference between P2-treated mice and control mice (Figure 5).

Mice were treated with 1000 mg/kg of P2 by i.p. or i.v. After 14 days’ observation, the mice were sacrificed, and five kinds of organs, including the heart, liver, spleen, lung and kidney, were excised. The organs were then fixed by paraformaldehyde and stained by H & E to make histopathologic analysis; magniﬁcation (200×, Nikon, Tokyo, Japan).

**Table 1:** Effects of P2 on the body weights of mice treated with P2 of maximum tolerated dose (MTD, 1000 mg/kg).

			**BA**	**AA**								
				1 day	2 days	3 days	4 days	5 days	6 days	7 days	10 days	14 days
Intraperitoneal injection	Male	Saline	18.68 ± 0.97	22.43 ± 1.06	22.34 ± 0.95	24.22 ± 1.39	25.22 ± 1.51	26.07 ± 1.89	28.22 ± 1.93	28.44 ± 2.02	32.53 ± 1.66	34.35 ± 1.76
		P2	18.52 ± 1.08	21.31 ± 0.78 *	21.00 ± 0.65 **	23.05 ± 0.84 *	24.75 ± 0.95	26.7 ± 1.05	28.24 ± 1.67	33.86 ± 1.42	33.86 ± 1.42	35.15 ± 1.56
	Female	Saline	19.55 ± 0.85	21.97 ± 1.12	21.63 ± 1.06	23.25 ± 1.31	24.16 ± 1.68	24.21 ± 1.58	25.38 ± 1.45	25.19 ± 1.50	26.82 ± 1.48	27.83 ± 1.40
		P2	19.31 ± 0.87	19.52 ± 1.07 **	19.47 ± 1.04 **	21.29 ± 1.24 **	23.12 ± 1.68	24.05 ± 1.60	24.82 ± 1.80	24.79 ± 1.71	27.47 ± 2.00	26.34 ± 2.64
Intravenous injection	Male	Saline	18.28 ± 0.94	23.54 ± 1.46	23.12 ± 1.31	25.16 ± 1.68	26.70 ± 1.77	28.19 ± 1.78	29.77 ± 1.84	30.40 ± 1.86	32.98 ± 1.95	34.52 ± 2.37
		P2	18.49 ± 0.82	21.17 ± 0.92 **	21.19 ± 0.90 **	23.41 ± 1.14 *	25.06 ± 1.01 *	26.48 ± 1.24 *	28.62 ± 0.94	29.50 ± 1.00	33.18 ± 1.33	33.72 ± 1.23
	Female	Saline	19.68 ± 0.77	22.75 ± 1.22	22.25 ± 1.21	23.79 ± 1.47	24.42 ± 1.59	24.87 ± 1.75	26.24 ± 1.47	26.25 ± 1.70	28.29 ± 1.79	29.47 ± 1.80
		P2	19.52 ± 1.03	20.81 ± 1.36 **	20.47 ± 1.31 **	22.23 ± 1.60 *	23.25 ± 1.37	23.94 ± 1.68	25.06 ± 1.62	24.87 ± 1.66	27.31 ± 1.66	28.62 ± 1.72

BA and AA stand for before administration and after administration, respectively; Values are expressed as mean ± SD (*n* = 10 mice per group). Data were analyzed by ANOVA followed by Student’s *t*-test; Significant difference between each treatment and the control are shown as *p* < 0.05 (*) and *p* < 0.01 (**).

**Table 2:** Antitumor effects of P2 against tumor growth on the S-180 and H-22 xenograft mice.

Models	Drug	Dosage (mg *·kg/day × 9)	Tumor weight (Mean ± SD, g)	Tumor growth inhibition (%)	Spleen index (mg/10 g)	Thymus index (mg/10 g)
S-180	Control	Normal Saline	1.53 ± 0.48	-	5.32 ± 0.97	2.94 ± 0.53
	CTX	25	0.30 ± 0.16 **	80.4	2.27 ± 0.91 **	1.04 ± 0.36 **
	P2	7	1.01 ± 0.35 **∆∆	34.0	8.29 ± 0.72 **∆∆	2.97 ± 0.66 ∆∆
		21	0.83 ± 0.43 **∆∆	45.8	9.22 ± 1.70 **∆∆	2.98 ± 0.62 ∆∆
		63	0.61 ± 0.27 **∆∆	60.1	10.58 ± 1.58 **∆∆	2.62 ± 0.57 ∆∆
H-22	Control	Normal Saline	1.40 ± 0.34	-	6.52 ± 1.35	2.99 ± 0.46
	CTX	25	0.39 ± 0.14 **	72.1	3.29 ± 1.03 **	1.56 ± 0.58 **
	P2	7	1.03 ± 0.29 **∆∆	26.4	7.83 ± 1.87 *∆∆	2.68 ± 0.48 ∆∆
		21	0.82 ± 0.29 **∆∆	41.4	9.28 ± 1.20 **∆∆	2.64 ± 0.45 ∆∆
		63	0.75 ± 0.25 **∆∆	46.4	11.20 ± 2.59 **∆∆	2.43 ± 0.66 *∆∆

Mice were injected with S-180 or H-22 subcutaneously into the right front armpit and randomly divided into five test groups. The mice were daily treated by P2 (7, 21, 63 mg/kg/day), CTX (25 mg/kg/day) or normal saline (NS, 10 mL/kg) by intravenous injection for nine consecutive days. The mice were sacriﬁced, and tumors were excised and weighed for evaluating the tumor growth inhibition. The spleen and thymus were also segregated and weighed to calculate the spleen index and thymus index. Cyclophosphamide (CTX) was used as the positive control. Data are presented as mean ± SD of 14 animals. Data were analyzed by ANOVA followed by Student’s *t*-test. Significant difference between each treatment and the control are shown as *p* < 0.05 (*) and *p* < 0.01 (**). Significant difference between each treatment and the CTX are shown as *p* < 0.01 (∆∆).

### 2.3. Evaluation of Antitumor *in Vivo*

**Figure 6:**
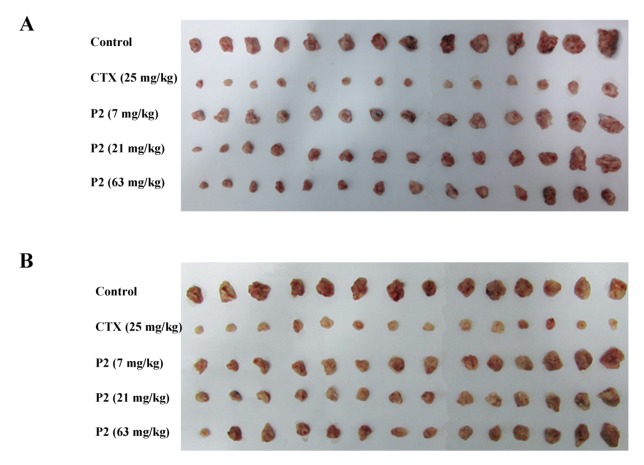
Antitumor effects of P2 against tumor growth on the S-180 (**A**) and H-22 (**B**) xenograft mice. Mice transplanted with S-180/H-22 cells were treated by P2 (7, 21, 63 mg/kg/day), CTX (25 mg/kg/day) or normal saline (10 mL/kg) for a consecutive nine days. Then the mice were sacrificed, and the tumors were excised. The images showed the tumor masses of mice treated by P2, CTX or normal saline.

To understand the inhibitory effect of P2 on the growth of tumor cells *in vivo*, mice hepatoma cells H-22 and sarcoma cells S-180 were selected to evaluate the *in vivo* antitumor effects of P2. The effects of P2 on mice transplanted with S-180 or H-22 are presented in Table 2 and Figure 6. The results revealed that P2 significantly decreased the tumor weights of S-180 tumor-bearing mice and H-22 tumor-bearing mice. The inhibitory rates were 34.0%, 45.8% and 60.1% for S-180 tumor-bearing mice and 26.4%, 41.4% and 46.4% for H-22 tumor-bearing mice at the dosages of 7, 21, 63 mg/kg/day, respectively. Furthermore, P2 could raise the spleen index, but had no obvious influence on the thymus index of the tumor-bearing mice (Table 2). 

## 3. Discussion

*A. subcrenata* contains various active ingredients, such as polysaccharides, amino acids, trace elements, vitamins and polypeptides, which showed good potential antitumor activity [3,21]. In our previous investigation, we had developed a fingerprint of 10 batches of antitumor active polypeptides from *A. subcrenata* by High Performance Liquid Chromatography (HPLC) [19]. In this study, *A. subcrenata* was treated under a series of processing, including tissue homogenation, high-speed centrifugation and gradient precipitation with ammonium sulfate to acquire the crude peptide extracts. The antitumor activities of peptides fractions were evaluated by MTT assay. To obtain the polypeptide being of best antitumor activity, the crude peptide extracts were further purified by DEAE-sepharose Fast Flow ion-exchange chromatography, and four fractions were collected. P2 was one of the fractions exhibiting excellent inhibitory effects on tumor cells.

In the clinic, many drugs have definite antitumor effects, but at the same time, patients often suffer from adverse side effects for the reason that the drugs are not specifically selective in cytotoxicity for tumor cells [22]. Many chemotherapy drugs affect the patients’ cardiovascular system, accompanied with heart failure, myocardial ischemia/infarction, hypertension, thromboembolism and arrhythmias, such as doxorubicin, clofarabine [23,24], as well as the immune system, accompanied with lipsotrichia, emaciation and hypoimmunity, such as cyclophosphamide [25,26]. In this paper, a range of testing results revealed that P2 had a good inhibitory capability on the growth of tumor cells *in vitro *and *in vivo* with little toxicity, which illuminated that P2 selectively inhibited the growth of the tumor cells.

In the preclinical antitumor drug-screening program, an extract that displayed an IC_50_ value below 30 μg/mL *in vitro* and a tumor growth inhibition rate above 30% *in vivo* was considered promising [27]. From the results of this study, we could get an idea that P2 might be one of the promising antitumor drugs. Scientists have revealed the underlying mechanisms of antitumor drugs, including direct anti-proliferation against tumor cell lines, such as inducing cells apoptosis [28] and indirect antitumor drugs, such as enhancing the immunity of the body to eliminate tumor cells, including stimulating lymphocyte transformation, enhancing phagocytic capability of peritoneal macrophages [29]. Our previous reports indicated that the polypeptides fraction from *A. subcrenata *could enhance immunity by promoting the proliferation of lymphocytes, enhancing the phagocytic ability of peritoneal macrophage (PMΦ) and the cytotoxicity of natural killer (NK) cells remarkably *in vitro* [30]. In this study**,** P2 displayed IC_50_ values much lower than 30 μg/mL on HeLa and HT-29 cells, which implied that the antitumor effects of P2 might be related to direct anti-proliferative effects *in vitro*. Meanwhile, P2 inhibited the growths of S-180 and H-22 tumors accompanied with increasing the spleen indexes remarkably, which suggested P2 also might exert the antitumor effects by enhancing the immunity of tumor-bearing mice.

## 4. Experimental Section

### 4.1. Chemical

Cyclophosphamide (CTX) was purchased from Shanxi Pude pharmaceutical Co., Ltd. China. Dimethyl sulfoxide (DMSO), 3-(4,5)-dimethylthiahiazo(-z-y1)-3,5-di-phenytetrazoliumromide (MTT), Penicillin G and streptomycin were obtained from Sigma Chemical (St. Louis, MO, USA). RPMI-1640 and Fetal Bovine serum (FBS) were purchased from GIBCO Invitrogen Corporation (San Diego, CA, USA). All other chemicals used were of analytical grade, available locally.

### 4.2. Extraction Procedure and Sample Preparation

*A. subcrenata *was purchased from Huangsha seafood market (Guangzhou, China). *A. subcrenata* was shelled and washed by distilled water three times. To obtain the active constituents, phosphate-buffered saline solution was added to *A. subcrenata *at 3:1 volume to weight ratio for homogenation and centrifugation to collect the supernatant. Ammonium sulphate powder was slowly added to the supernatant to precipitate the protein components. The precipitate was collected for further purification by ion-exchange chromatography eluted with sodium chloride solution of different concentrations. The effluent fractions were detected by a protein detector at 280 nm. The effluent fraction of P2 was concentrated and dialyzed. A dry powder of P2 was obtained by vacuum freeze-drying.

### 4.3. Cell Lines and Culture

Cell lines used for evaluation of the *in vitro* cytotoxicity in this study included seven tumor cell lines and one normal cell line, namely HeLa (cervical cancer cell line), HT-29 (colon cancer cell line), HepG2 (hepatocellular carcinoma cell line), K562 (leukemia cell line), DU145 (prostate cancer cell line), SPC-A-1 (lung adenocarcinoma cell line), B16-F10 (murine melanoma cell line) and L-02 (human normal liver cell line). All of cell lines were provided by the Shanghai Institutes for Biological Sciences, Chinese Academy of Sciences, and the cells were cultured in RPMI-1640 medium, which was supplemented with 10% heat-inactivated fetal bovine serum, 100 U/mL penicillin and 100 U/mL streptomycin and cultured in an atmosphere of 5% CO_2_ at 37 °C. Cells were collected for the experiments in the logarithmic growth phase.

The cell lines used for evaluation of the *in vivo* antitumor activity in this study included mice sarcoma S-180 and hepatoma cells H-22, which were provided by Shanghai Institutes for Biological Sciences, Chinese Academy of Sciences. S-180 ascite and H-22 ascite were maintained *in vivo* in mice by transplantation of 0.2 mL of ascites (1 × 10^7^ cell) from the infected mice to the non-infected mice.

### 4.4. Animals

Kunming mice (KM mice, 20 ± 2 g) were purchased from Experimental Animal Center of Sun Yat-sen University (Guangzhou, China). Mice were housed in a temperature (23 °C) and humidity (55%)-controlled room on a 12 h light/dark cycle and given free access to water and food.

### 4.5. Cytotoxicity Assay

For the antitumor screening tests, P2 was dissolved and diluted to 1.56, 6.25, 25, 100, 400 and 1600 μg/mL by RPMI-1640 medium. For the tests of time-dependent and dose-dependent effects, the concentrations of P2 were 0.5, 1, 2, 4, 8, 16, 32, 64 and 128 μg/mL. The HeLa and HT-29 cells were treated with different concentrations of P2 for 24, 48 and 72 h. For the cytotoxicity on normal cells, human liver L-02 cells were treated with P2 for 48 h. All tests were performed by MTT assay according to the paper [31], with some necessary variation. Briefly, cells were seeded in the well of 96-well microtiter plates and incubated with different concentrations of P2 for a setting time. After that, 20 μL of MTT (5 mg/mL) was added to each well, and then, the plates were incubated for another 4 h at 37 °C. The supernatant was aspirated and MTT-formazan crystals were dissolved in 200 μL of DMSO. Absorbance was measured spectrophotometrically at 570 nm. Cell growth inhibition was evaluated by comparing the absorbance of treated and untreated cells. The percentage of cell growth inhibition was calculated as the following formula:

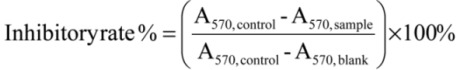
(1)


### 4.6. Acute Toxicity Testing

Eighty mice were randomly divided into four groups of either of the sexes. Two groups of mice received a single dose of P2 at 1000 mg/kg. The sample was administered via the caudal vein and abdominal cavity (0.2 mL/10 g). The control groups were given normal saline solution (0.9% NaCl). Mouse survival and body weight variations were monitored daily for 14 days. After 14 days’ observation, mice were sacrificed, and five kinds of organs, including the heart, liver, spleen, lung and kidney, were excised. The organs were fixed by paraformaldehyde and stained by hematoxylin and eosin (H & E) to make a histopathologic analysis. The MTD was defined as the highest dose that induced no more than 15% weight loss comparing with control, caused no toxic death and no visible organ lesions [32].

### 4.7. Antitumor Activity *in Vivo*

Animal care and treatment were performed at Jinan University’s experimental animal center. A total of 70 mice (male, body weight 20 ± 2 g) were injected with 6 × 10^6^ cells/mouse of S-180 (sarcoma-180) or H-22 (mice hepatoma cells) subcutaneously into the right front armpit. 24 h after implantation of tumor cells, the mice were randomly divided into five test groups with 14 mice per cohort. The mice were daily treated by P2 (7, 21, 63 mg/kg/day), CTX (25 mg/kg/day) or normal saline (NS, 10 mL/kg) for nine days. The mice were sacrificed, and the tumors were excised and weighed for evaluating the tumor growth inhibition at 24 h after the end of treatment. The spleen and thymus were also segregated and weighed to calculate the spleen index and thymus index for assessing the effects of P2 on the immune system of the tumor-bearing mice. The tumor inhibitory rate and organ index were calculated by the following formulae:

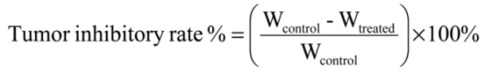
(2)

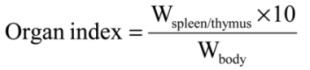
(3)
W_control _and W_treated_ were the average tumor weights of the control and treated mice, respectively. W_spleen/thymus_ and W_body _stand for the average weights of spleen/thymus and body of the mice. 

### 4.8. Statistical Analysis

Data are shown as mean ± SD. Statistical analyses were performed using an unpaired, two-tailed Student’s *t*-test. All comparisons were made relative to untreated controls and significant difference is indicated as * *p* < 0.05 and ** *p* < 0.01.

## 5. Conclusions

In conclusion, the present study demonstrates the potent antitumor properties of P2, a polypeptide fraction from *A. subcrenata*, and its toxicity. P2 displayed good antitumor activity in dose-dependent and time-dependent manners, with low toxicity *in vitro *and *in vivo*. Further studies would focus on the elucidation of the mechanism of action and the isolation of its active constituents.
